# Clinical and Deep-Learned Evaluation of MR-Guided Self-Supervised PET Reconstruction

**DOI:** 10.1109/TRPMS.2024.3496779

**Published:** 2025-03

**Authors:** Jessica B. Hopson, Sam Ellis, Anthime Flaus, Colm J. McGinnity, Radhouene Neji, Andrew J. Reader, Alexander Hammers

**Affiliations:** Department of Biomedical Engineering, King’s College London; Department of Biomedical Engineering, King’s College London; King’s College London & Guy’s and St Thomas’ PET Centre, King’s College London; King’s College London & Guy’s and St Thomas’ PET Centre, King’s College London; Department of Biomedical Engineering, King’s College London; Department of Biomedical Engineering, King’s College London; King’s College London & Guy’s and St Thomas’ PET Centre, King’s College London

**Keywords:** Self-supervision, Image Reconstruction, Image Quality Assessment, PET imaging, PET-MR imaging

## Abstract

Reduced dose Positron Emission Tomography (PET) lowers the radiation dose to patients and reduces costs. Lower count data, however, degrades reconstructed image quality. Advanced reconstruction methods help mitigate image quality losses, but it is important to assess the resulting images from a clinical perspective. Two experienced clinicians assessed four PET reconstruction algorithms for [^18^F]FDG brain data, compared to a clinical standard reference (Maximum-Likelihood Expectation-Maximization (MLEM)), based on seven clinical image quality metrics: global quality rating, pattern recognition, diagnostic confidence (all on a scale of 0-4), sharpness, caudate-putamen separation, noise, and contrast (on a scale between 0-2). The reconstruction methods assessed were a guided and unguided version of self-supervised maximum *a posteriori* EM (MAPEM) (where the guidance case used the patient’s MR image to control the smoothness penalty). For 3 of the 11 patient datasets reconstructed, post-smoothed versions of the MAPEM reconstruction were also considered, where the smoothing was with the point-spread-function used in the resolution modelling. Statistically significant improvements were observed in sharpness, caudate-putamen separation, and contrast for self-supervised MR-guided MAPEM compared to MLEM. For example, MLEM scored between 1-1.1 out of 2 for sharpness, caudate-putamen separation and contrast, whereas self-supervised MR-guided MAPEM scored between 1.5-1.75. In addition to the clinical evaluation, pre-trained Convolutional Neural Networks (CNNs) were used to assess the image quality of a further 62 images. The CNNs demonstrated similar trends to the clinician, showing their potential as automated standalone observers. Both the clinical and CNN assessments suggest when using only 5% of the standard injected dose, self-supervised MR-guided MAPEM reconstruction matches the 100% MLEM case for overall performance. This makes the images far more clinically useful than standard MLEM.

## I Introduction

**A**N important tool in the diagnosis of memory clinic patients is Positron Emission Tomography (PET) with [^18^F]FDG. PET allows for functional imaging of the brain, and when incorporated into a hybrid simultaneous PET – magnetic resonance imaging (PET-MR) imaging system, can also provide complementary structural or multiparametric imaging [1]. However, one limitation that prevents hybrid PET-MR from being used earlier in the diagnostic pathway is that a radiation dose needs to be administered [2]. Not only does this have associated radiation risks for both the staff and patient, but additionally, it is expensive to manufacture the radioligand according to the Good Manufacturing Practice standards [3].

One method of overcoming this limitation is to use a reduced radiation dose during acquisition [4]. However, traditional clinical standard PET reconstruction methods, such as maximum-likelihood expectation-maximization (MLEM) [5] and ordered-subset expectation-maximization (OSEM) [6] are susceptible to image degradation at lower doses [7]: with an increasing number of iterations of the reconstruction algorithm, the noise is amplified [8], [9], degrading image quality [4]. The motivation of this work is therefore to determine if novel reconstruction methods can be used clinically to mitigate these limitations of traditional reconstruction methods at lower doses.

A problem with using MLEM and OSEM is the level of regularization required is dependent on the level of noise in the PET data, and so the noise needs to be regularized during the reconstruction process. Traditional methods of analysis regularization for PET reconstruction involve applying a penalty term in the objective function [10]. One group of such novel reconstruction methods is the maximum *a-posteriori* EM (MAPEM) family. Within this family of methods, the patient’s MR image can guide the PET image during reconstruction [11]. The kernel expectation-maximization method (KEM) exploits the MR image to derive kernels, regularizing the image noise [12]. KEM, using 10% of the total PET data, has shown comparable performance to MLEM at a 100% dose level [12].

However, more recently, there has been a shift to use artificial intelligence for PET reconstruction [10], [13]. Deep learning can also be used to optimize reconstruction hyperparameters [14], and means that no hyperparameter needs to be set by the user. If PET images generated by deep learning from lower-dose PET data can be deemed of an acceptable quality, then this may speed up clinical translation of low-dose imaging [15]. One example of using deep learning to regularize PET reconstructions are generative adversarial networks (GANs). GANs have outperformed KEM for PET reconstruction [16]. Another novel approach is bootstrap-optimized self-supervised reconstruction, whereby no external training data is needed and no hyperparameter selection is required, but only the penalty term or prior is user-defined [17], being used as a regularization strategy [18]. This outperformed standard MLEM PET reconstruction [17], [18]. The self-supervised approach uses a bootstrapped sample of the measured PET data, and finds the regularization strength needed for a regularized image update based on the bootstrapped data to best match the unregularized update from the original measured data (the only reason for a difference in these updates is assumed to be due to noise in the data). The hyperparameter found necessary for agreement between the update based on the bootstrapped data and the update based on the original measured data is then regarded as the regularization necessary to compensate for noise in the data, and hence that regularization strength is subsequently used to update the image from the original measured data.

Whilst novel reconstruction methods have been evaluated analytically, clinical assessment is also important to ensure that such reconstruction methods can be used in a clinical setting. To the best of the authors’ knowledge, there has been little investigation into the viability of novel reconstruction methods from the viewpoint of a clinician. One study by Mehranian *et al*. [19] showed that MR-guided methods may preserve diagnostic image quality of PET-MR brain images, acquired using 10% of a standard radioligand dose, for the metrics of global quality rating (GQR), pattern recognition (PR) and diagnostic confidence (DC). This current study continues that of Mehranian *et al*. [19], by including different MR-assisted methods, such as bootstrap-optimized MR-guided self-supervised MAPEM [18]. Additional clinical metrics are also evaluated in this current study, including metrics for sharpness, caudate-putamen separation (CP), contrast and noise.

One of the drawbacks with the clinical evaluation of medical images is the associated time and organizational commitments, especially when obtaining readings from multiple experienced clinical observers [20], and thus also slows large-scale reconstruction investigations as clinical evaluations are not immediate. Therefore, it is important to be able to automate image quality assessment. It was shown that this is possible by Hopson *et al*. [21], where a convolutional neural network (CNN) pre-trained on ImageNet [22] allowed for improved performance in the predicting clinical evaluations of OSEM [^18^F]FDG brain PET images, compared to training from scratch. This work builds upon that presented in Hopson *et al*. [21] and uses the principle of transfer-learning to automatically predict the clinical evaluations of novel reconstruction techniques.

There are two aims of this work. Firstly, to compare novel MAPEM-based self-supervised reconstruction methods to a clinical standard method to determine any potential for clinical translation. Secondly, investigate whether CNNs have the potential to be treated as automated standalone observers for analyzing the quality of novel reconstruction methods.

## II Materials and Methods

### A Clinical Patient Dataset

The dataset came from 11 memory clinic patients (6 female and 5 male; average ages of 60.5 ± 7.2 and 56.2 ± 7.3, respectively) with suspected dementia, who were a part of a larger study. In this study, each patient underwent both an [^18^F]FDG PET-CT scan acquired on a Discovery 710 PET-CT (General Electric, Chicago, USA) and an [^18^F]FDG PET-MR scan acquired on a Biograph mMR simultaneous PET-MR (Siemens Healthineers, Erlangen, Germany), at the King’s College London & Guy’s and St Thomas’ PET Centre, as part of a wider study. The dataset was acquired by Dr Colm J. McGinnity and [^18^F]FDG was used as the radioligand in both the PET-CT and PET-MR scans. The median injected dose was 221 ± 35.9 MBq (approximately equivalent to 8 mSv [23]). The PET-CT acquisition commenced 31 ± 3 minutes post-injection, with an acquisition time of 15 minutes. Only the PET-MR scans were used in this work and were acquired an average of 98 minutes after injection, with a 30 minute acquisition duration. Throughout this study, some images were scored by two separate clinicians, and some images were scored only by a single clinician (Section II.D). This was due to time-constraints associated with reading the images. All of the images were assigned a random five-letter code, and presented to the clinician in alphabetical order, so as to provide a representative, non-biased sample. Therefore, due to this randomness, the images read by a single clinician were the ones which were presented in the time available.

### B Standard Reconstruction Method

All reconstructions were carried out using 100% and 5% of the counts, with the 5% achieved by resampling the binned sinograms. For comparison to standard reconstructions, all datasets were reconstructed at two separate dose levels (using 5% and 100% of the total counts) using the maximum-likelihood expectation-maximization (MLEM) algorithm. For MLEM reconstruction, 63 iterations were used with a 2.5 mm point-spread-function (PSF), simulating the non-PSF projectors used in standard clinical reconstruction Siemens e7 tools, when using an in-house reconstruction software (APIRL [24]). The images were post-smoothed with a Gaussian filter with FWHM of 4 mm to correspond to the clinical standard. Of the 78 doubly read images (Section II.C), 22 of the images were reconstructed using MLEM.

### C Self-Supervised Reconstruction Methods

#### 1) Unguided Self-supervised MAPEM

A self-supervised MAPEM algorithm, based on a method self-regularized via data bootstrapping [17], was used. The algorithm was run over 1000 iterations automatically setting the penalty strength when given a penalty function. For self-supervised “unguided” MAPEM, an isotropic quadratic penalty, *U(****θ****)*, was used (1):

(1) $$\text{U}(\theta )=\frac{1}{4}\overset{J}{\underset{j=1}{\sum }}\underset{k\in N_{j}}{\sum }w_{jk}{\left(\theta_{j}-\theta_{k}\right)}^{2}$$

This considers the pair-wise intensity difference between each voxel, *j*, and each of its neighbors, *k*. Here, **θ** is the image vector. All weights *w*_*jk*_, were set to a value of 1/27 for a neighborhood size of 3 × 3 × 3. A PSF resolution modelling kernel of 4.5 mm full-width at half maximum was used for this reconstruction (unguided MAPEM). For the smoothed version of this reconstruction algorithm, a final post-reconstruction smoothing of 4.5 mm was applied. When PSF modelling is used in the reconstruction, a post-smoothing with the same kernel amounts to a change of the spatial basis functions to the PSF kernel.

In total, for images that were scored by two clinicians, 22 reconstructions were carried out using this method without post-reconstruction smoothing, with an additional 6 reconstructions with post-reconstruction smoothing. For the singly-read images (Section II.D), a further 12 images without post-reconstruction smoothing, and another 11 images with post-reconstruction smoothing, were used.

#### 2) MR-guided Self-supervised MAPEM

The second self-regularized, self-supervised MAPEM algorithm was the “guided” version, where the weights given in (1) were not uniform, but were calculated from the corresponding MR image [25], using (2) and (3):

(2) $$w_{jk}=\exp\left(\frac{-∥f_{j}-f_{k}{∥}^{2}}{2\sigma_{f}^{2}}\right)\exp\left(\frac{-∥r_{j}-r_{k}{∥}^{2}}{2\sigma_{s}^{2}}\right)$$

Feature vectors, **f**_j_ for the voxel, *j*, and **f**_k_ for each of its neighbors, *k*, are the voxel values in the MR image, (i.e. 1D features) and the neighborhood size is 3 × 3 × 3. The first kernel is based on feature vectors (patches from the prior [^18^F]FDG image), with σ_f_ = 1 which controls kernel width. The second kernel uses σ_s_ = 4 mm, defined as the spatial distance between ***r***_*k*_.and ***r***_*j*_, where ***r***_*j*_ is the spatial location of voxel, *j*, and ***r***_*k*_ is the spatial location of its neighbors, *k*. Features were normalized by the standard deviation of each element across the image (3):

(3) $$f_{j,m}=\frac{{\hat{f}}_{j,m}}{SD_{m}\left({\left\{{\hat{f}}_{j}\right\}}_{j=1}^{J}\right)}$$

Where ${\hat{f}}_{j,m}$ are the unnormalised feature vectors and $SD_{m}\left({\left\{{\hat{f}}_{j}\right\}}_{j=1}^{J}\right)$ is the standard deviation of the *m*^*th*^ element of the feature vectors over all voxels. For each voxel, the 7 highest weights were used, with all others set to 0, as per “largest value sparsification” used by Bland *et al*. [9] and the Bowsher prior [26]. Similarly to the unguided version, the guided method also had a post-reconstruction smoothed version, achieved by smoothing with the PSF kernel (4.5mm) post-reconstruction, with the same rationale.

In total, for images that were scored by two clinicians, 22 reconstructions were carried out using this method without post-reconstruction smoothing, with an additional 6 reconstructions with post-reconstruction smoothing. For the singly-read images (Section II.D), a further 13 images without post-reconstruction smoothing, and another 13 images with post-reconstruction smoothing, were used.

**Fig. 1:**
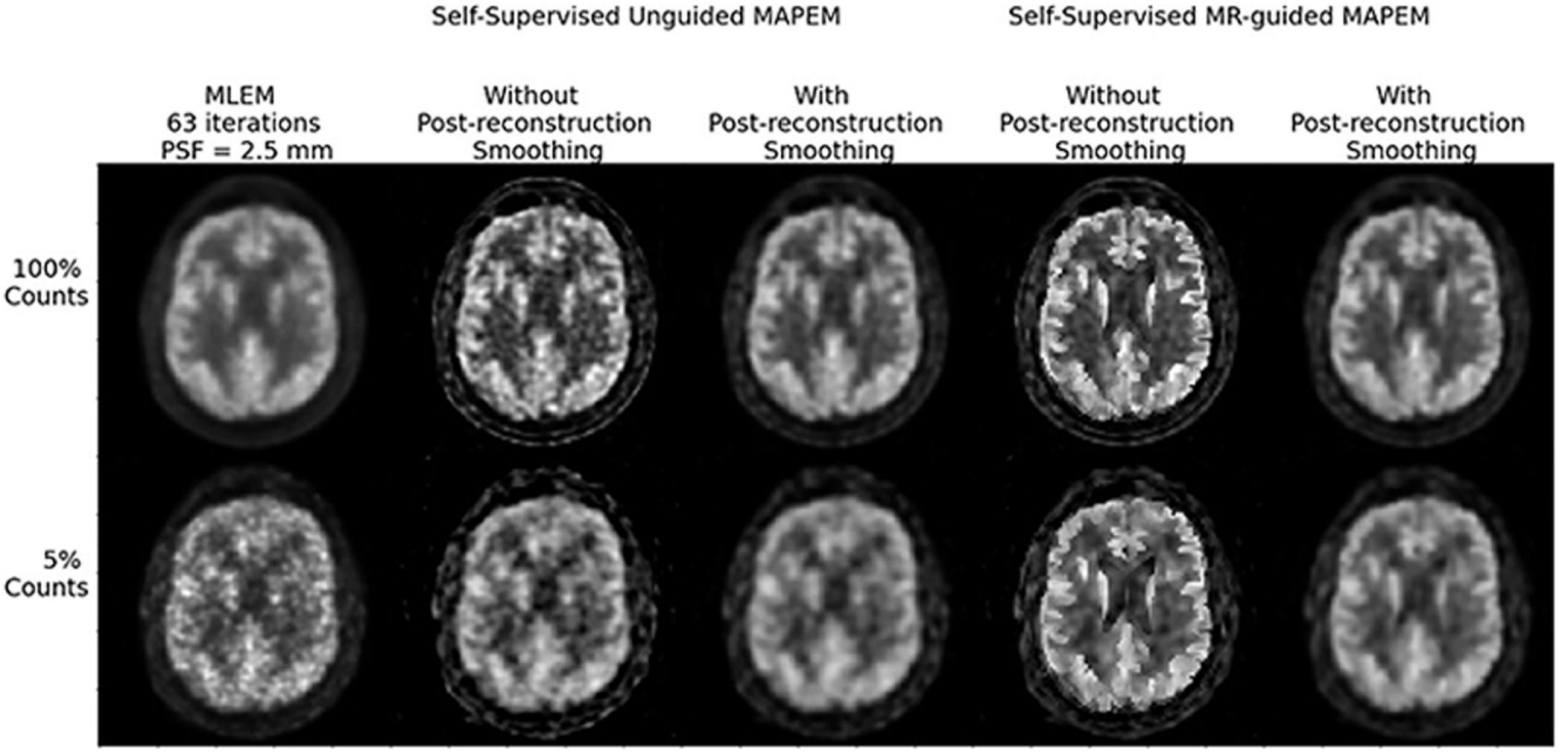
Example cropped slices of each of the reconstruction methods at two different dose levels (5% and 100%). From left to right, the reconstruction method becomes increasingly complex, with the left-most column standard MLEM.

Post-reconstruction smoothing for both MAPEM methods was applied to the last 3 of the 11 patients obtaining 78 reconstructions from 11 subjects, which were read by two clinicians. Figure 1 shows a cropped example slice from each reconstruction method.

### D Evaluation Methods

Seven clinical metrics were designed to determine the driving force of the image quality. Table I shows the definitions and scoring scales for each metric. All images were blinded and randomized prior to clinician scoring. The GQR, PR and DC were designed to be subjective quality metrics, whilst the metrics of sharpness, CP, contrast and noise were designed to incorporate more technical aspects of the reconstructions. The image quality metrics also included a rating for “better than clinical standard” to determine whether these reconstruction methods have the potential to outperform the clinical standard OSEM/MLEM for future clinical implementation. For sections III.A and III.B, all reconstructions were scored by two experienced independent clinicians; for 6 out of the 7 metrics, there was an agreement within 0.5 for over 70% of the scores, which is within label noise. For comparison, the overall performance score was calculated (the average over all 7 individual clinical metrics). A score of 4 for GQR, PR and DC was only available for the last 30 reconstructions.

The second evaluation method was automatic image quality assessment CNNs, based on that used in Hopson *et al*. [21]. However a DenseNet169 backbone [27] instead of a VGG16 backbone was used. This was because when transfer learning to the images in this task, DenseNet169 achieved a lower training error. The input to this CNN were 2D patches of size 80 × 80 from OSEM images at varying dose levels, initially trained to predict GQR, PR and DC. As in [21], random patches from each image were used. These 2D patches were extracted from each of the three anatomical planes such that the whole 3D image was used in to make the prediction. The median of the predictions were taken to get an overall prediction for each image volume. However, to use this model for the task outlined in this section, the projection head was removed and replaced with another to predict the output of the 7 clinical quality scores. All weights in the model were then unfrozen, and the learning rate was reduced to 10^-5^, to fine-tune the model, updating the weights for the new clinical task. The new model was trained using two examples from each method and dose level, such that 20 images were used in the training dataset, from two patients. The validation dataset consisted of all 10 reconstruction methods (one example per method) from a single patient. In total, three cross-folds were used to validate this model. For the CNN studies, a total of 62 images (from 9 independent patients), which had been read by one clinician, were used for testing. A score of 4 for GQR, PR and DC was also available for the extra 62 singly-read reconstructions used for the CNN. Table II summarizes the patient information for the images that were read by two separate clinicians, including reconstruction method and how each patient was used in the CNN study. Table III summarizes the patient information for the images that were read by a single clinician only.

## III Results

### A Analytical Assessment of Clinical Evaluations

**Fig. 2:**
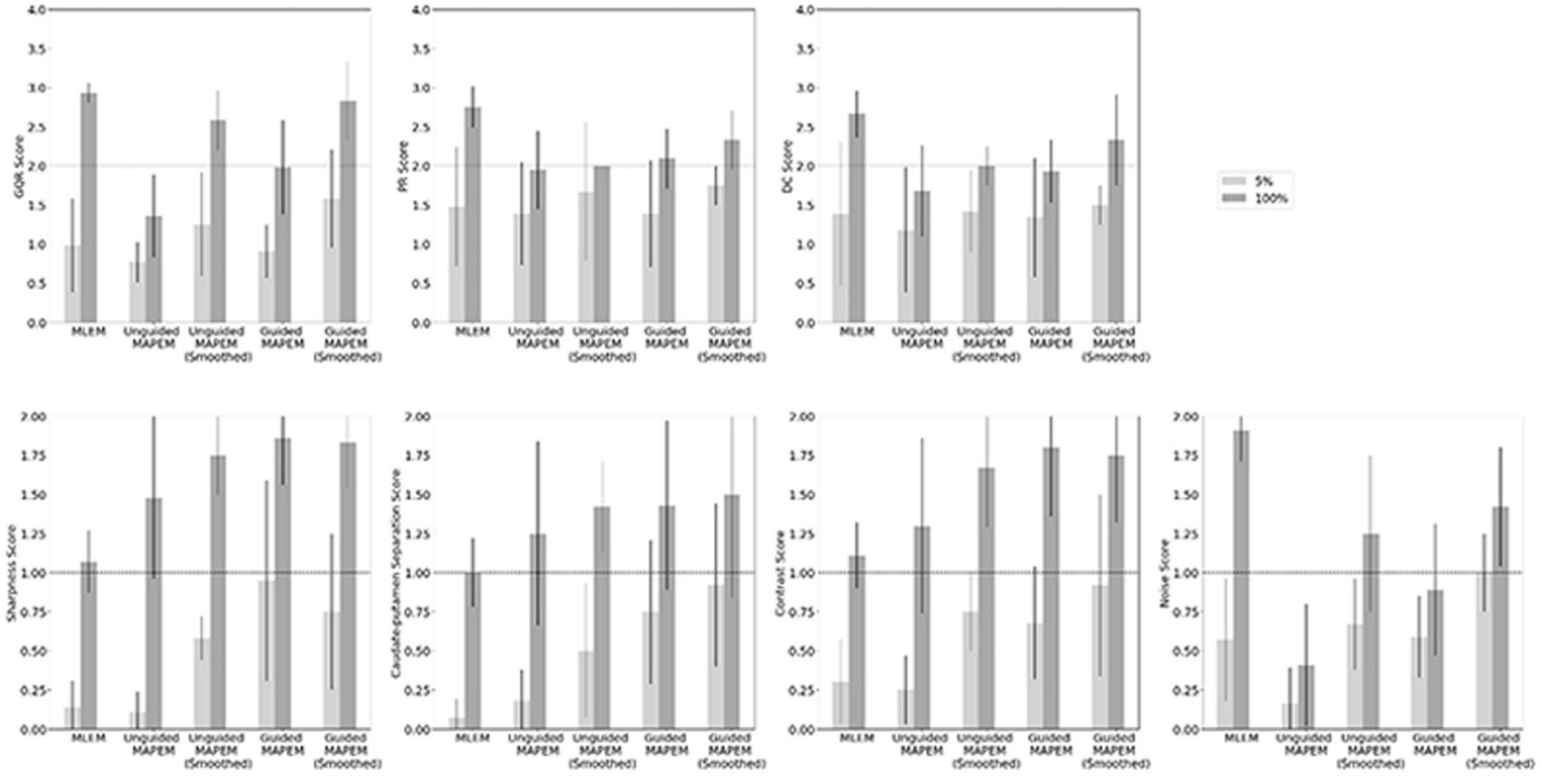
Average clinical scores between two clinicians of the 78 doubly read images. The light grey bars show the scores for the 5% count level, whilst the darker grey bars shows the scores for the 100% count level. Each error bar is the standard deviation for each method. The dashed line indicates the score required for acceptable use in a clinical setting. For each of these metrics, the higher the score, the better the reconstruction in terms of that specific metric. See Table I for definitions and scores.

Figure 2 shows the bar charts of the average scores of the 78 images scored by two clinicians, for each of the 7 image quality metrics. The error bars are the standard deviations, and the dashed line shows the point at which the reconstruction was judged to be of the usual clinical quality. Throughout this work, this threshold is defined as ≥ 2 for GQR, PR and DC, and ≥ 1 for sharpness, CP, contrast and noise. Comparing the reconstruction methods using 100% of a standard injected dose (dark grey bars in Fig. 2), MLEM performed the best for the more subjective metrics of GQR, PR and DC. Apart from unguided MAPEM for the three subjective metrics, and guided MAPEM for DC, all reconstruction methods at 100% reach the threshold for clinical usability. With increasing complexity of the reconstruction method (from left to right), the more technical metrics of sharpness, CP and contrast increased in scores, with the MR-guided reconstruction methods providing the highest rating. Apart from the metric of noise, however, MLEM is the worst performing reconstruction method for the more technical metrics of sharpness, CP and contrast. Conversely, for noise, only the smoothed, but not the unsmoothed, 100% MAPEM reconstructions reach the minimum clinical threshold whereas MLEM performs the best. For all seven image quality metrics, MR-guided MAPEM with post- reconstruction smoothing at 100% was amongst the highest performing reconstruction methods, consistently reaching the threshold for clinical viability.

At 100% of the standard injected dose, all methods reached the threshold for being clinically useable for the three of the four more technical metrics (sharpness, CP and contrast). Only the MR-guided MAPEM with post-reconstruction smoothing reached this threshold at 5% for noise, and came close for the other technical metrics. Of the more technical metrics, three (sharpness, CP and contrast) were scored consistently higher for the MAPEM methods than for the standard MLEM. The biggest improvement in clinical rating compared to MLEM was seen for the self-supervised MR-guided MAPEM reconstructions with post-smoothing, especially for sharpness, CP and contrast. For example, MLEM achieved a score of 0.07 ± 0.12 for CP at a 5% count level, compared to 0.92 ± 0.52 for the equivalent using the self-supervised MR-guided MAPEM method. Fig. 2 showed in all cases, the 100% reconstructions were scored higher than for the 5% reconstructions. However, at a 5% count level, self-supervised MR-guided MAPEM with post-reconstruction smoothing was deemed at least “poor but useable” or of a “clinical standard” for 6 out of the 7 metrics, reaching the clinical viability threshold for noise, compared to only 3 out of the 7 metrics for MLEM. Both smoothed MAPEM method versions generally outperformed the non-smoothed versions, especially for noise, GQR, PR and DC. Unguided MAPEM performed superiorly compared to MLEM for sharpness, CP and contrast, but inferiorly for the other metrics.

**Fig. 3:**
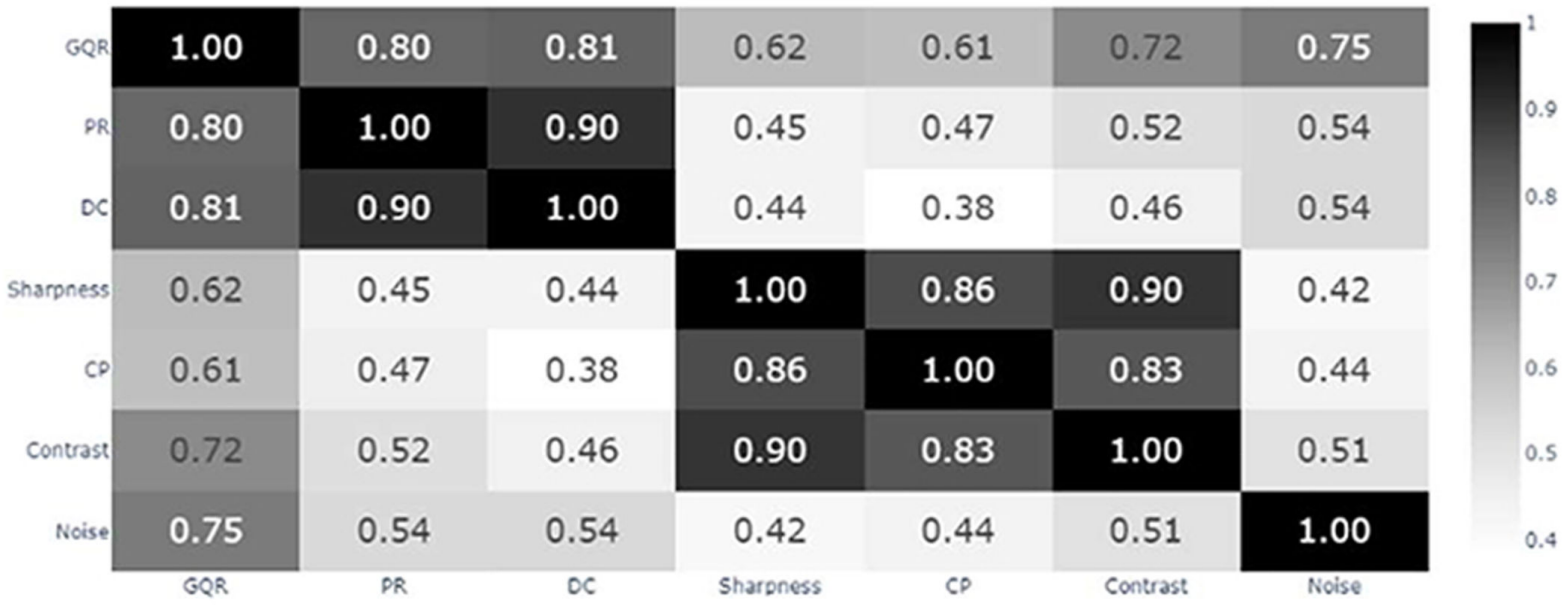
Correlation matrix of all metrics for the doubly-scored 78 images. Values are the Spearman’s correlation coefficient. GQR = global quality rating; PR = pattern recognition; DC = diagnostic confidence; CP = caudate-putamen separation.

Fig. 3 shows the correlation matrix for all metrics (using the Spearman’s correlation coefficient). The more subjective metrics (GQR, PR and DC) were strongly positively correlated with one another. For 3 out of the 4 more technical metrics (sharpness, CP and contrast), there was a strong positive correlation. “Noise”, however, correlates more with GQR, PR and DC, than with the other more technical aspect metrics.

### B Statistical Analysis of Clinical Evaluations

A Wilcoxon signed-rank test was used to test for statistical significance of the different ratings between the self-supervised methods and MLEM (Table IV). While MLEM was superior for GQR, PR and DC over both MAPEM methods without post-reconstruction smoothing, no significant difference could be determined for the smoothed variants. For most of the more technical ratings of sharpness, CP and contrast, both MR-guided variants were rated significantly superior to MLEM.

**Fig. 4:**
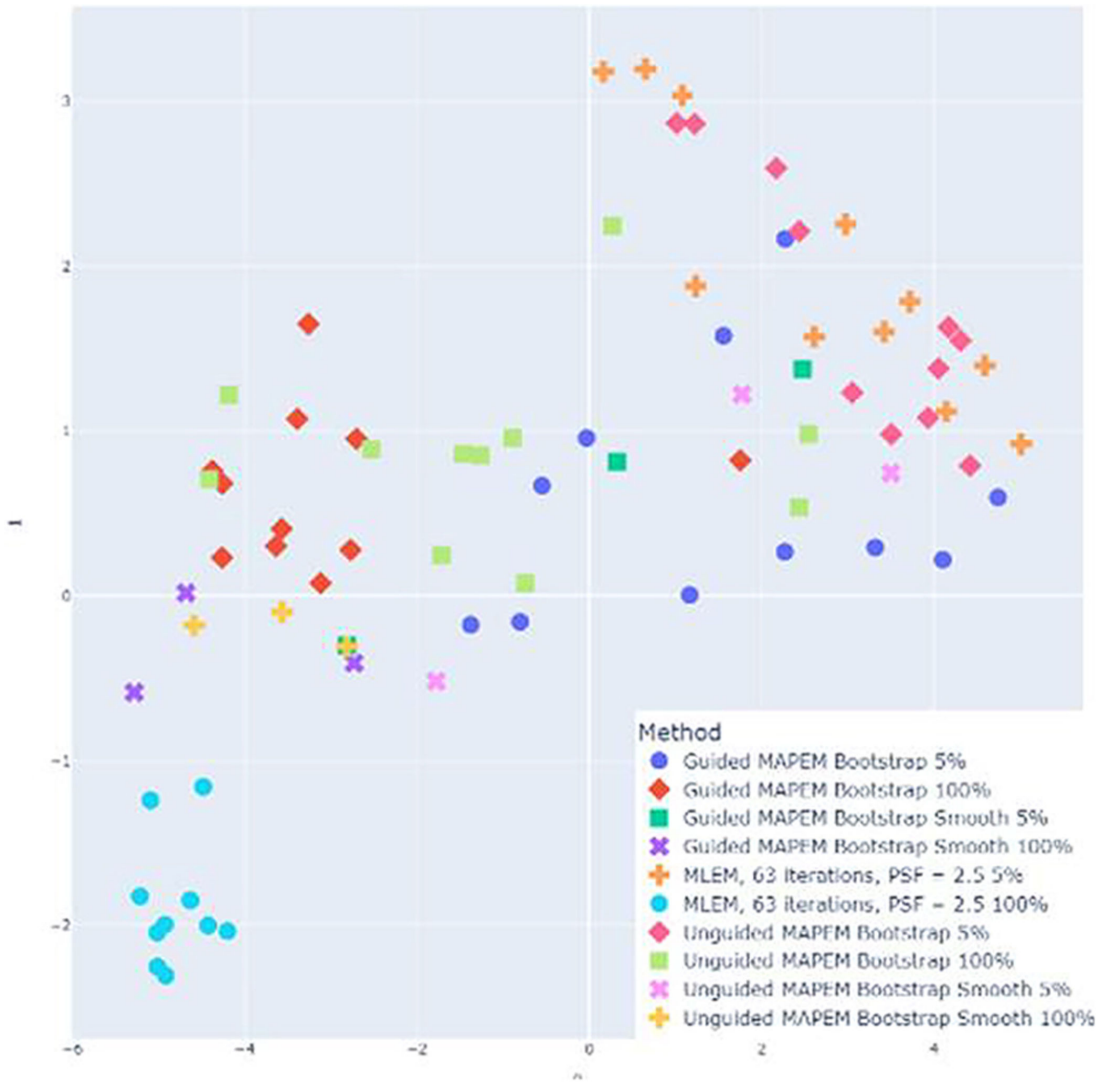
A t-distributed stochastic neighbor embedding (t-SNE) visualization for the 78 images that were doubly-scored.

A t-distributed Stochastic Neighbor Embedding (t-SNE) visualization [28] (Fig. 4) was also produced to determine the presence of any clustering of the methods in terms of the clinical score. This statistical visualization method is used as a dimensionality reduction technique, such that high-dimensional data can be displayed as a two-dimensional data point. In this case, this method was chosen to preserve the 7-dimensional information of the clinical scores, projected to a 2-dimensional space for visualisation, and to observe any clustering of the scores for the same reconstruction methods. This method models the probability distribution of neighbours (a set of points which are closest to each point) around each point, by calculating the Euclidean distances. This is modelled as a Gaussian distribution in the high-dimensional space, and as a t-distribution in the 2-dimensional space. The algorithm provides a mapping between the high- and 2-dimensional spaces that minimises the differences between the two spaces.

### C Deep Learning Models as Independent Observers

**Fig. 5:**
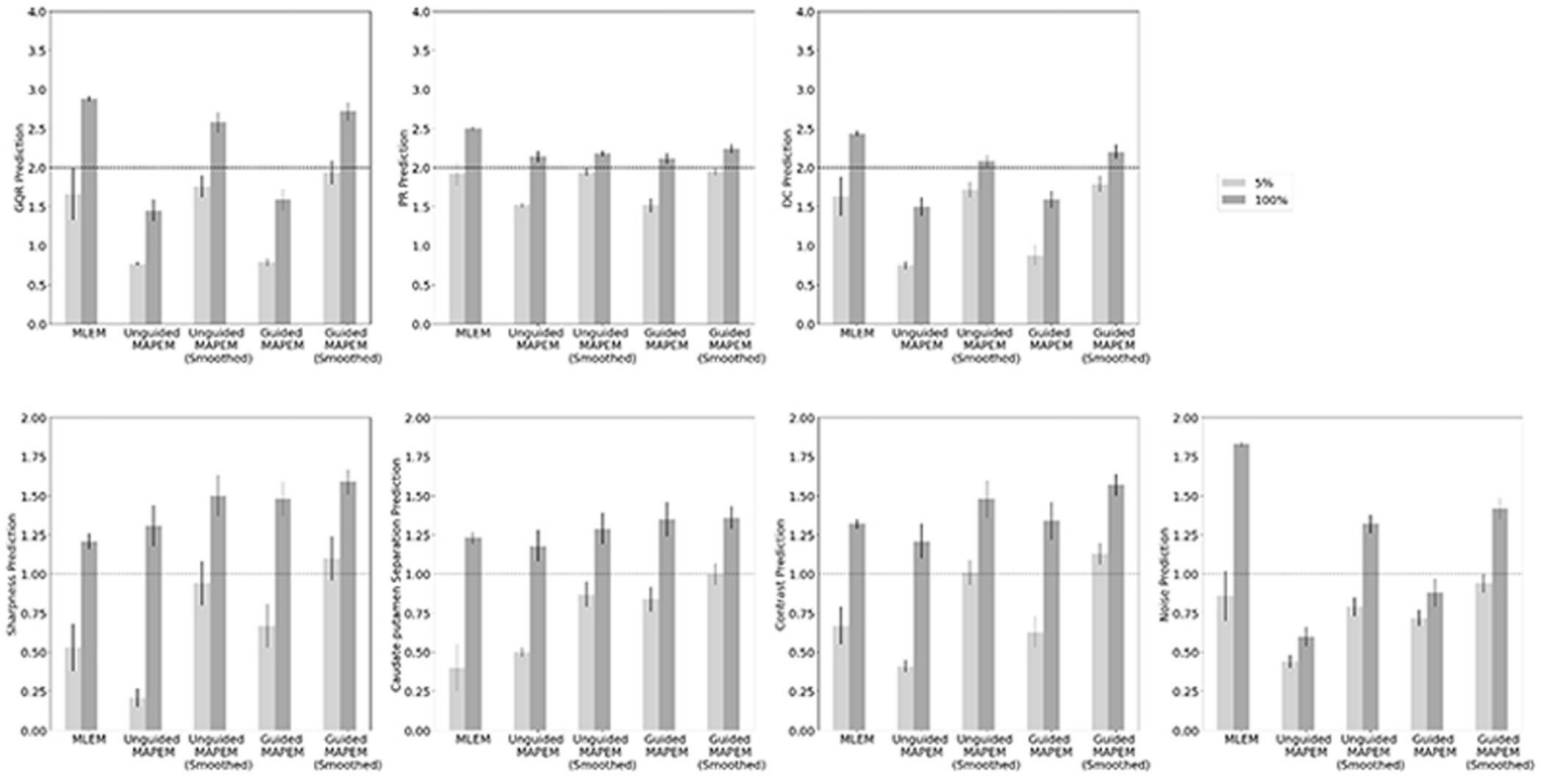
Bar charts of the CNN predictions for each of the seven quality metrics at both 5% (light grey) and 100% (dark grey) of counts for each of the 7 clinical metrics. The error bars are the standard deviations calculated between reconstructions. The dashed line indicates the score required for usability in a clinical setting.

This section concerns only the predictions of the CNN based on the 62 singly-read images. These images were separate to the 78 used in Sections III.A and III.B, as the scale was extended for GQR, PR and DC to include a “better than clinical standard” score, and were read by one clinician (a direct comparison between the deep-learned and human observers is shown in sections III.D - III.F). Fig. 5 shows the average predictions given by the deep ensemble (i.e. 3 cross-folds) of CNN observers for each of the 7 quality metrics for both 5% (light grey) and 100% (dark grey) count reconstructions. Fig. 5 is the equivalent of Fig. 2, which concerns the human observers. For the more subjective metrics (first three panels), MLEM, unguided MAPEM and MR-guided MAPEM, both with post-reconstruction smoothing, consistently reached the threshold for clinical viability at 100%, with all reconstruction methods reaching this threshold for PR at 100%. For the more technical metrics (last four panels), MR-guided MAPEM with post-reconstruction smoothing, even at a 5% count level, reached the clinical usability threshold for sharpness, CP and contrast, whilst 5% unguided MAPEM with post-reconstruction smoothing met this threshold for contrast. Generally, the more complex the reconstruction (to the right in Fig. 5), the better the performance for both doses for sharpness, CP and contrast.

### D Comparison of Low-Count Reconstructions

**Fig. 6:**
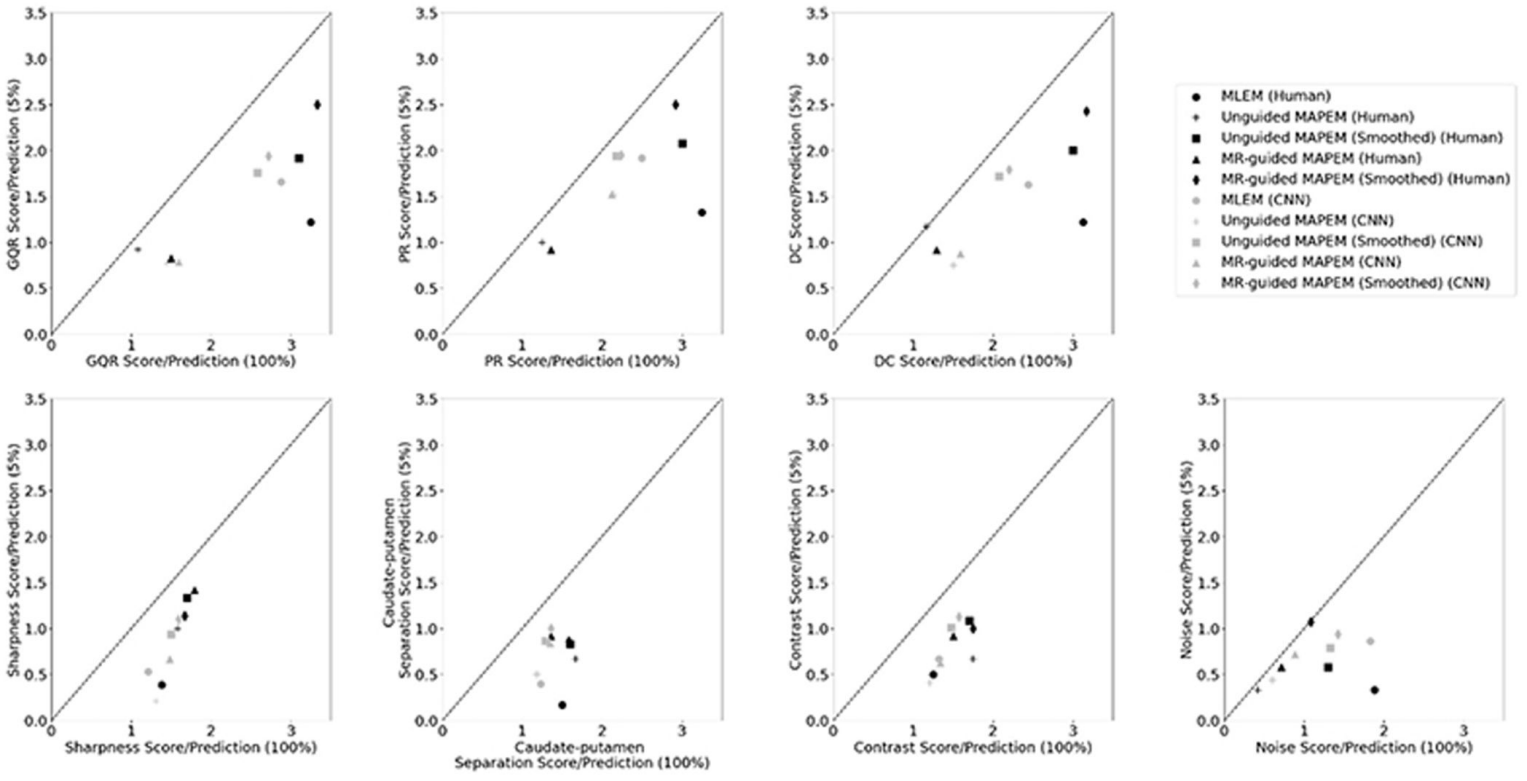
Scatter plot of the image quality assessment scores for both the human clinical scores and the CNN predictions, with the 100% count level case plotted against the 5% count level case, for each of the seven metrics. The average across patients for each reconstruction method is taken. The identity line is indicated by the dashed black line.

To determine the clinical usability of the 5% reconstruction cases, scatter plots for each metrics for the 100% cases were plotted against the 5% cases, for both the human and CNN scores for the additional 62 images that were singly read (Fig. 6). Each point represents the average score for the specific reconstruction method. Generally, for most reconstruction methods, the points lie below the identity line (i.e. 100% scores > 5% scores). MR-guided MAPEM with post-reconstruction smoothing for noise, and unguided MAPEM without post-reconstruction smoothing for DC were exceptions when scored clinically, as the 5% and 100% cases were deemed identical.

### E Comparison between Human and CNN Observers

**Fig. 7:**
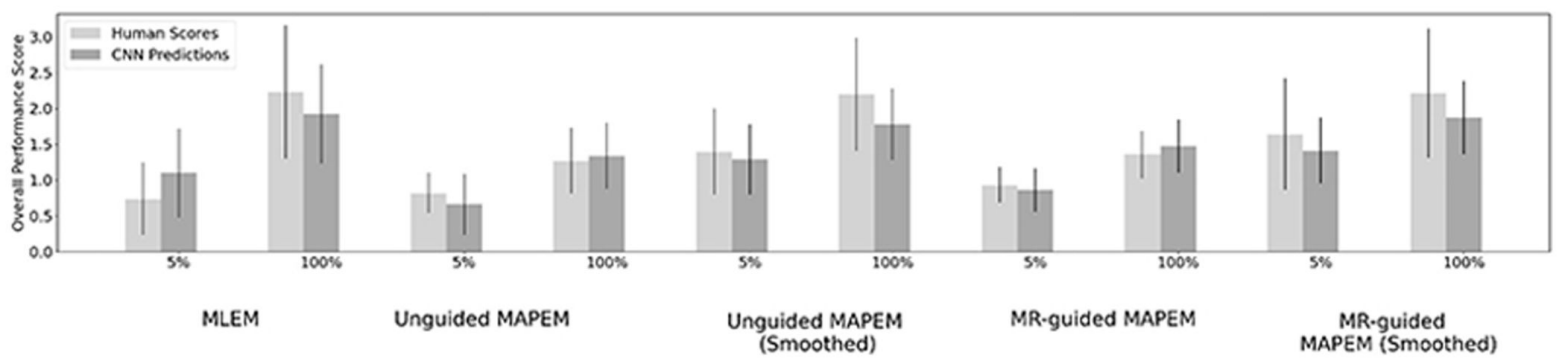
Overall performance score between the human clinical scores and the ensemble of CNN observers. The overall performance score was obtained by taking the average of all 7 metrics for each reconstruction method. The error bar is the standard deviation across all metrics.

The deep-learned observer was more likely than the human observers to provide similar scores for each of the reconstruction methods, especially for PR (Fig. 6). This was evident through the clustering of points, which was seen to a lesser degree for the clinical score counterparts. For example, all of the CNN predictions for PR sat between the minimum and maximum human scores, suggesting a much narrower range of predictions by the deep-learned observers than the human clinical scores. Fig. 7 shows the overall comparison score across all metrics for each reconstruction method at two different dose levels. The CNN and human observers both followed similar trends and their standard deviations overlapped for every reconstruction method. For example, both predicted the MLEM, unguided MAPEM with post-smoothing, and MR-guided MAPEM with post- reconstruction smoothing at 100% to be amongst the best performing methods, and unguided MAPEM without post- reconstruction smoothing and MLEM at 5% to be amongst the worst performing methods. Both the deep-learned and human observers showed that MR-guided MAPEM with post-reconstruction smoothing at 5% matched MLEM at 100% and outperformed it at 5%. The CNN predictions (Fig. 7) tended to be slightly lower than the human clinical ratings, for 7 out of the 10 reconstruction methods, but were higher for the remaining 3 reconstruction methods. Comparing the scores given by the CNN and the clinician for individual reconstructions, there was an exact agreement between both observers for 3 out of the 4 metrics, for full-count MR-guided MAPEM with post-reconstruction smoothing and unguided MAPEM with post-reconstruction smoothing at 5%. The largest agreement discrepancy between both observers was for GQR for MR-guided MAPEM without post-reconstruction smoothing reconstructed using 5% of the counts: the human observer rated all images of a clinical standard, but the deep-learned observer rated all images below clinical standard.

### F Overall Comparison between all Reconstruction Methods

The 100% cases were consistently rated higher than the 5% cases for overall performance (Fig. 7). The overall performance score for full-count MLEM was similar to full-count MR-guided MAPEM with post-reconstruction smoothing and full-count unguided MAPEM with post-reconstruction smoothing. Similarly to Fig. 2, Fig. 7 showed MLEM at 5% was rated lower by both the human and deep-learned observers, compared to MR-guided MAPEM with post-reconstruction smoothing. Of the images singly scored by the clinician, no 5% MLEM reconstruction was rated above a clinical standard for CP, whilst 33% of reconstructions were rated to be of a clinical standard for PR and DC. The CNN observer agreed with the human observer, only rating 33% of MLEM images at 5% at or above a clinical standard for PR, which was the highest percentage. Low-count MR-guided MAPEM with post-reconstruction smoothing improved the number of clinically useable images (judged by the human observer) for contrast, from 22% for MLEM to 86%, and from 0% (MLEM) to 100% for the CNNs, the latter matching that for full-count MLEM.

## IV Discussion

For sharpness, CP and contrast (the more technical metrics), MLEM was consistently outperformed by the other four novel reconstruction methods investigated (Fig. 2). By comparing the image quality metrics at a 5% count level (light grey bars in Fig. 2), MLEM (the clinical standard) was among the worst performing reconstruction algorithms across all quality metrics. This suggests that the current clinical standard method cannot be used clinically with injected doses reduced to 5% of the normal level, as it is subject to image degradation [7]. A 5% count level was chosen based on prior work suggesting reasonable feasibility in principle [21], [29]. While even lower doses may be technically feasible, in clinical practice, the aim is to obtain robust and reliable data in a single patient visit, and we felt 5% was a reasonable level to aim for. Clinically, 5% is an ambitious lower-bound, but at intermediate count levels (e.g. 10 – 25%), this reconstruction method may have potential to be clinically translated especially for lower doses.

In practice, a patient otherwise injected with, for example, 221 MBq (equating to approximately 8 mSv [23]), would only receive 11 MBq (equating to approximately 0.4 mSv [23]). The patient and staff would benefit from receiving a lower radiation dose. As less tracer would be required, there is also the potential for economic savings via reduced tracer costs and via longer scanning hours with the same initial tracer dose produced or procured. Similarly, brain research could benefit through cost savings and in particular by making the scanning of healthy controls more acceptable. However, MR-guided MAPEM with post-reconstruction smoothing generally outperforms MLEM at the 5% count level, suggesting that this method is not as susceptible to image degradation as MLEM at lower counts. This may therefore allow for easier clinical translation of this reconstruction method as intermediate count levels may be clinically acceptable. In contrast, MLEM only reached this clinical usability threshold for 2 of the 7 image quality metrics at a 5% count level. MR-guided MAPEM with post-reconstruction smoothing is rated statistically significantly better than MLEM for 3 out of the 7 metrics (Table IV). The original paper that proposed bootstrap-optimized MR-guided MAPEM [17], suggested that they outperformed MLEM, which is confirmed in this study. Thus, self-supervised MR-guided MAPEM with post-reconstruction smoothing may potentially be used clinically, including at reduced dose levels.

For GQR, PR and DC, full-count MLEM achieved a score of ~3 (“good/excellent”) for each metric (Fig. 2). MLEM is widely used as the clinical standard, and therefore clinicians were likely more confident in making their assessments. Such a bias towards the familiar also explains why the more subjective metrics GQR, PR and DC were scored higher for MLEM compared to the more technical aspects. Applying a post-reconstruction smooth to both self-supervised reconstruction methods improved performance of clinical assessments (Fig. 2), as the noise level was reduced [30], helping clinicians assess the images. MLEM performs statistically significantly better than both unguided and MR-guided MAPEM without post-smoothing (Table IV and Fig. 2), suggesting that post-reconstruction smoothing may be key for clinical translation.

Fig. 3 showed a potential explanation for the relation between metrics in Fig. 2. GQR, PR and DC had a strong correlation with one another and summarized all aspects of the image quality: if a clinician deemed the image of a useable quality (GQR), then they were more likely to detect pathological patterns (PR) and were more confident in making a diagnosis (DC). Both clinicians noted that when reading images with subtle findings (or normal images), high quality images were needed for a confident diagnosis to be made. However, it was also noted that clinicians were able to make a confident diagnosis on lower-quality images if the pathology was clear, for example, a clear Alzheimer’s disease pattern. Generally, the clinicians agreed with one another across the different reconstruction methods on the clinical diagnosis of the patients. Sharpness, CP and contrast were very strongly correlated: a sharper image has a greater resolution and contrast [31], and likewise greater separation between the caudate and putamen. Thus, a reconstruction method that performed well on one of these three metrics tended to perform well on the other two. Conversely, “Noise” was more strongly correlated with GQR, PR and DC than with sharpness, CP and contrast, suggesting noise was important for GQR, PR and DC ratings.

The t-SNE visualization (Fig. 4) suggested clinicians could more easily differentiate MLEM from other reconstruction methods, likely due to their familiarity and bias towards MLEM. A lower level of clustering is seen for the other methods, indicating the clinicians found it harder to differentiate between methods.

Scores derived from the CNNs followed similar trends to the clinical readings. The 100% cases were predicted to have higher ratings than the 5% cases. MLEM reconstruction had a much lower score for the more technical metrics bar noise (sharpness, CP and contrast) than the novel reconstruction methods. In agreement with the clinical scorers, the CNN ratings also suggested potential for clinical use of self-supervised MR-guided MAPEM with post-reconstruction smoothing, as it was consistently rated by the CNNs as amongst the best performing reconstruction algorithms (Fig. 5). These findings are in agreement with those in sections III.A and III.B, and shows that the CNN could be used as an automated standalone observer, alleviating time burdens, and providing instant assessments for large-scale reconstruction investigations [20].

For all metrics, a higher score indicated a better performance. The CNNs appeared able to differentiate between the 5% and 100% cases across all methods. As expected, the lower-count reconstructions were more likely to be scored lower [19]. However, the CNN was less likely to differentiate between methods in the case of PR, shown by the increased clustering compared to the clinical scores (Fig. 6), suggesting a limitation of using the current CNN for the subjective metrics. Another limitation of using the current CNN for assessment of image quality is that it may not be generalizable to other reconstruction methods or dose levels. To overcome this, further examples would need to be given to the CNN of different reconstruction methods to improve its ability to generalize. A study by Zhang *et al*. [32] also used a DenseNet CNN to predict image quality assessment of PET images on a 5-point scale. They found that when reducing down to two groups (“poor” and “high” image quality), the CNN was able to differentiate between the two groups with a sensitivity and specificity of 0.91 and 0.80, respectively. Another example is by Pfaehler *et al*. [33]. In this study, the task was to classify 30 oncological (mostly upper body) PET images acquired on a PET-CT scanner as “EARL compliant” (an image quality benchmark) or not, then subsequently classify the images as meeting the older or newer EARL standards. The CNNs, trained from scratch (i.e. no pre-training), correctly identified 100% of EARL1 compliant images, and 85% of EARL2 compliant images. The authors cite this automated image quality assessment as eliminating the need for manual region-of-interest definition, as well as the ability to apply the model to multiple imaging sites. The CNN in the current work is also able to differentiate between the 5% and 100%, which is akin to “poor” or “high” quality images, broadly comparable to “EARL compliant” or not. It may be argued that more classical metrics, such as signal-to-noise ratio, and the metrics designed in this work, may be adequate to differentiate between high and low image quality, without the need for a CNN. Whilst this is true, for example, the findings in Fig. 1 show that this is possible, using a CNN can help overcome the time constraints on clinicians, making large-scale reconstruction investigations much faster.

However, one of the main limitations of this work, is that there is a small sample size used in training the CNN, with only one tracer used. To overcome this, different tracers could be used, along with data from more patients, to further improve generalization to unseen images, for example, from different imaging centers, which is a recurring problem in CNN studies for medical imaging [34]. Thus, even though generalizability may need to be improved, the examples show that there is merit of using CNNs in automating PET image quality assessments. Using 5% of the standard injected dose, MR-guided MAPEM with post-reconstruction smoothing was shown to outperform MLEM, when assessing the clinical usability of individual reconstructions, for the more technical metrics. This was also evident with the more subjective metrics, where the percentage of images deemed to be of a clinical standard for MR-guided MAPEM with post-reconstruction smoothing outperformed MLEM at 5%, and matched full-count MLEM for GQR and PR in terms of clinician scoring. MLEM images at 5% of the counts were more likely to be deemed clinically useable for the subjective metrics compared to the more technical metrics, due to the clinicians’ bias towards MLEM. However, the clinicians agreed that with more exposure to images reconstructed with self-supervised MR-guided MAPEM with post-reconstruction smoothing, then this would be preferential in the future. There were more discrepancies between the deep-learned observer and human observer for the 5% cases, perhaps due to more variation in clinical scores at lower-doses for OSEM images, used as the basis of the CNN observers.

The main aim of Fig. 7 was to show the relative ranking of the reconstruction methods given by both the clinician and the CNN. This metric allows for all of the seven clinical image quality metrics to be incorporated to determine the overall score given to each reconstruction method. Both the clinician and CNN showed the same relative ranking of the reconstruction methods. Despite the deep-learned observer providing lower scores across several reconstruction methods, the standard deviations overlapped, suggesting the CNNs predicted within the range of the clinician, showing their potential as automated standalone observers. These results in Fig. 7 were generated from a single observer, but were in line with those from 2 clinicians (Fig. 2).

The overall performance score took into account all clinical metrics. Whilst most reconstruction algorithms were deemed clinical viable for the 100% cases, MLEM, MR-guided MAPEM and unguided MAPEM both with post-reconstruction smoothing were among the best performing. This was expected from MLEM, due to the familiarity bias. Out of all algorithms using 5% of counts, MR-guided MAPEM with post-reconstruction smoothing performed best (Fig. 7), suggesting potential clinical viability, including for low-count cases.

## V Conclusion

This work determined the clinical viability of novel reconstruction algorithms reconstructed with both 5% and 100% of the counts. Bootstrap-optimized self-supervised MR-guided MAPEM with post-reconstruction smoothing consistently outperformed MLEM, and the clinicians agreed that this method would be preferable over MLEM with more training using these images, especially for low-count reconstructions. The other aim was to determine if a CNN could be used as an automated standalone observer, mitigating the burden on clinicians when assessing images for large-scale reconstruction investigations, whilst providing a rapid automated assessment. This deep-learned observer predicted image quality scores within the range of the human observer and followed similar trends, showing their potential as automated standalone observers. Future work may include additional transfer-learning experiments to further improve CNN predictions, and comparison to other reconstruction methods. Further validation of these methods could be carried out by applying them to different datasets, such as epilepsy patients.

## Figures and Tables

**Table I:** Definitions And Scoring Scales Of The 7 Clinical Image Quality Metrics

Metric	Definition	Scoring Scale
Global Quality Rating (GQR)	Aesthetic component of the reconstruction, taking into account qualities such as noise level and resolution	0 – “unacceptable” 1 – “poor but useable” 2 – “acceptable” 3 – “good/excellent” 4- “better than clinical standard”*
Pattern Recognition (PR)	Determines the presence of any pathological patterns suggesting a particular diagnosis	
Diagnostic Confidence (DC)	The certainty with which the clinician can use the reconstruction to observe any patterns and make a diagnosis	
Sharpness	The clarity of the image, and the level of definition within the image	0 – “poor” 1 – “clinical standard” 2 – “better than clinical standard”
Caudate-putamen separation	The level of distinguishability between the caudate and putamen	
Contrast	The contrast between grey and white matter structures, evaluated in all three orientation with the cursor placed in the parasagittal mid-cingulate gyrus	0 – “poor grey matter-white matter differentiation” 1 – “clinical standard PET” 2- “better than clinical standard”
Noise	Based on the uniformity of the white matter	0 – “poor” 1- “intermediate/acceptable noise” 2 – “homogeneous/low noise”

**Table II:** Patient Information For Those Datasets Read By Two Separate Clinicians

Patient Number	Diagnosis	Total Number of Prompts at a 100% Injected Dose Level (millions. 3 s.f.)	Reconstruction Method	Number of Clinicians that Read the Images	Images Reconstructed and Scored	How images were used in the CNN
1	Alzheimer’s disease	317	MLEM Unguided MAPEM Guided MAPEM	2	Reconstructed and scored at both 5% and 100% count levels	Training
3	Primary progressive aphasia (Alzheimer’s disease)	140	MLEM Unguided MAPEM Guided MAPEM			
5	Normal	250	MLEM Unguided MAPEM Guided MAPEM			
6	Non-degenerative/vascular	120	MLEM Unguided MAPEM Guided MAPEM			
10	Normal	353	MLEM Unguided MAPEM Guided MAPEM			
15	Normal	230	MLEM Unguided MAPEM Guided MAPEM			
17	Alzheimer’s disease	405	MLEM Unguided MAPEM Guided MAPEM			
25	Alzheimer’s disease	469	MLEM Unguided MAPEM Guided MAPEM			
27	Normal	74.6	MLEM Unguided MAPEM Unguided MAPEM Smoothed Guided MAPEM Guided MAPEM Smoothed			Training/cross-fold validation
28	Normal	315	MLEM Unguided MAPEM Unguided MAPEM Smoothed Guided MAPEM Guided MAPEM Smoothed			
29	Normal	720	MLEM Unguided MAPEM Unguided MAPEM Smoothed Guided MAPEM Guided MAPEM Smoothed			

**Table III:** Patient Information For Those Datasets Read By One Clinician Only

Patient Number	Diagnosis	Total Number of Prompts at a 100% Injected Dose Level (millions, 3 s.f.)	Reconstruction Method	Number of Clinicians that Read the Images	Images Reconstructed and Scored	How images were used in the CNN
32	Normal	563	Unguided MAPEM Smoothed	1	Reconstructed and scored at both 5% and 100% count levels	Testing
			MLEM		Reconstructed and scored at 5%	
			Unguided MAPEM Guided MAPEM Guided MAPEM Smoothed		Reconstructed and scored at 100%	
37	Normal	573	MLEM Unguided MAPEM Guided MAPEM		Reconstructed scored at both 5% and 100% count levels	
			Guided MAPEM Smoothed		Reconstructed and scored at both 5%	
38	Antiphospholipid syndrome	483	Guided MAPEM Smoothed		Reconstructed and scored at both 5% and 100% count levels	
			MLEM Guided MAPEM		Reconstructed scored at 5%	
			Unguided MAPEM Smoothed		Reconstructed and scored at 100%	
43	Normal	811	MLEM Unguided MAPEM Guided MAPEM Guided MAPEM Smoothed		Reconstructed and scored at both 5% and 100% count levels	
			Unguided MAPEM Smoothed		Reconstructed and scored at 5%	
48	Normal	442	MLEM Unguided MAPEM Unguided MAPEM Smoothed Guided MAPEM Guided MAPEM Smoothed		All methods reconstructed and scored at 5% 100% count levels	
51	Normal	893	Guided MAPEM Guided MAPEM Smoothed		Reconstructed and 100% count levels	
			MLEM Unguided MAPEM Smoothed		Reconstructed and scored at 5%	
54	Normal	514	MLEM Unguided MAPEM		Reconstructed and scored at both 5% and 100% count levels	
			Unguided MAPEM Smoothed Guided MAPEM Guided MAPEM Smoothed		Reconstructed and scored at 5%	
57	Antiphospholipid syndrome	683	MLEM Unguided MAPEM Guided MAPEM Guided MAPEM Smoothed		Reconstructed and scored at both 5% and 100% count levels	
			Unguided MAPEM Smoothed		Reconstructed and scored at 100%	
60	Normal	669	Unguided MAPEM Smoothed		Reconstructed and scored at both 5% and 100% count levels	
			MLEM Unguided MAPEM		Reconstructed and scored at both 5%	
			Guided MAPEM		Reconstructed scored at 100%	
64	Alzheimer’s disease	512	Unguided MAPEM Unguided MAPEM Smoothed Guided MAPEM Smoothed		Reconstructed and scored at both 5% and 100% count levels	
			MLEM		Reconstructed and scored at 5%	
			Guided MAPEM		Reconstructed and scored at 100%	

**Table IV:** Wilcoxon Signed-Rank Test For Comparison To Mlem. “Sig.” significant difference; Italics: MLEM is statistically better; bold: self-supervised method is superior; “c.d.” cannot determine (due to too few samples, as post-reconstruction smoothing was applied to 3 of the 11 patients); “n.s.” not significant.

Method	GQR	PR	DC	Sharpness	CP	Contrast	Noise
Self-supervised Unguided MAPEM	*Sig.*	*Sig.*	*Sig.*	n.s.	n.s.	n.s.	*Sig.*
Self-supervised Unguided MAPEM (Smoothed)	c.d.	n.s.	n.s.	**Sig.**	c.d.	**Sig.**	n.s.
Self-supervised MR-guided MAPEM	*Sig.*	*Sig.*	*Sig.*	**Sig.**	**Sig.**	**Sig.**	**Sig.**
Self-supervised MR-guided MAPEM (Smoothed)	c.d.	c.d.	n.s.	**Sig.**	**Sig.**	**Sig.**	n.s.

## Data Availability

The patient data supporting this article can only be shared in anonymized form due to the type of participant consent obtained; please contact the corresponding author if required.
